# Measuring global health inequity

**DOI:** 10.1186/1475-9276-6-16

**Published:** 2007-10-30

**Authors:** Daniel D Reidpath, Pascale Allotey

**Affiliations:** 1 Centre for Public Health Research, Brunel University, Uxbridge, UK

## Abstract

**Background:**

Notions of equity are fundamental to, and drive much of the current thinking about global health. Health inequity, however, is usually measured using health inequality as a proxy – implicitly conflating equity and equality. Unfortunately measures of global health inequality do not take account of the health inequity associated with the additional, and unfair, encumbrances that poor health status confers on economically deprived populations.

**Method:**

Using global health data from the World Health Organization's 14 mortality sub-regions, a measure of global health inequality (based on a decomposition of the Pietra Ratio) is contrasted with a new measure of global health inequity. The inequity measure weights the inequality data by regional economic capacity (GNP per capita).

**Results:**

The least healthy global sub-region is shown to be around four times worse off under a health inequity analysis than would be revealed under a straight health inequality analysis. In contrast the healthiest sub-region is shown to be about four times better off. The inequity of poor health experienced by poorer regions around the world is significantly worse than a simple analysis of health inequality reveals.

**Conclusion:**

By measuring the inequity and not simply the inequality, the magnitude of the disparity can be factored into future economic and health policy decision making.

## Introduction

Inequity fuels the fire of moral outrage. It is justifiably and acutely observable in the area of global health. Global health researchers describe it, theorise about it, and look for solutions to it [1-4]. In all these endeavors however, there is a discomfort between knowing that inequity exists, "knowing" that it is a significant problem, and being able to say just how big a problem it is. At least a part of the difficulty arises from the unclear relationship between global health inequality and global health inequity.

Health inequality refers simply to the uneven distribution of health in or between populations. Furthermore, some health inequalities are unavoidable [5]. Never can the situation arise in which an entire population has the same (i.e., equal) health status [6]. Nonetheless, health inequalities should be of particular interest when those inequalities are attributable to determinants that fall within the capacity of people and societies to moderate. When these kinds of disparities occur, the issue becomes one of health inequities – not simply unevenness but unfairness in the distribution of health. Health inequities may be thought of as the presence of systematic disparities in health (or its social determinants) between more and less advantaged social groups [[7], p.256, [8]].

Poverty is one such social determinant of health, possibly the major social determinant, which is readily (if reluctantly) amenable to human intervention. It is strongly associated with health outcomes globally, and differences in population health that are patterned by economic capacity are frequently highlighted as examples of health inequity [9,10].

It is a relatively straightforward matter to measure the inequality in the global distribution of health. Measures of health inequality include population attributable risk, rate ratios, rate differences, and the concentration index [11-14]. But just how unfair is a health inequality?

Consider the ratio of health outcomes for the highest and lowest socioeconomic *n*-tile as a measure of health inequality. This produces such observations as a 5.3 times greater chance of under-five mortality among the poorest quintile of Peruvians as for the richest quintile [[15], p.11]. Similar statistics may be observed between the health outcomes of the rich and the poor all over the globe [16]. Indeed, a quick perusal of any recent *World Health Report *will show a continuing and strong relationship between national wealth and national health [17-19].

There are difficulties, however, with any health equity analysis that is based on a statistic of health inequality [7,11,12]. Measures such as the concentration index, the population attributable risk, or rate ratios, each implies a one-to-one correspondence between the magnitude of the inequality and the magnitude of the inequity. Although each statistic may represent a sound empirical measure of health inequality, it does not necessarily capture the normative aspects of the distributive unfairness of that inequality [16]. In the absence of equity specific measures, even among those acutely aware of the difference between measuring an inequity and measuring an inequality [20,21], the distinction is often lost.

Economically determined health inequalities are not simply unfair because societies have the capacity to redress them. They are additionally unfair because the burden that is experienced by a society in virtue of the population's health is itself mediated by the population's wealth. Wealthy regions have a greater capacity to support poor health than poor regions, and this means that the impact of poor health on both the individual sufferer and the society is significantly less. Wealthy regions tend to have invested in (and have the ongoing capacity to invest in) better physical infrastructure, healthcare services, and social services, all of which mitigate the impact of mortality and morbidity [22,23]. This means that any unfairness associated with the uneven distribution of health is not limited simply to the distribution of health itself.

It might reasonably be argued, therefore, that in an analysis of health equity, the variation in population health should be weighted by the wealth of the populations under examination. This is grounded in the idea that, in a fair world, those with the least economic capacity to overcome the encumbrances of poor health should be those who are most protected from it. Richer regions are in a position to bear a greater health burden than poor regions, because the richer regions have a greater economic capacity to overcome the additional encumbrances imposed by that poor health. Although health cannot, in reality, be redistributed between populations, a counter factual analysis based on this notion provides an insight into the actual magnitude of the inequity. A similar approach is taken by Gravelle (1998) in his "artefactual argument" for the redistribution of individual income [24].

Using global burden of disease data from the World Health Organization (WHO), the magnitude of health inequity in the regional distribution of health was examined by contrasting a new measure of health inequity (a wealth-weighted measure of health inequality) against a regular measure of health inequality.

## Methods

The equity of the distribution of health status was examined using data from the 14 global mortality sub-regions of the WHO [18,25]. The six WHO regions were Africa (AFRO), the Americas (AMRO), Eastern Mediterranean (EMRO), Europe (EURO), South East Asia (SEARO), and the Western Pacific (WPRO). Each region was further subdivided into between two and four sub-regions according to the adult and child mortality profile of country clusters: very low child and low adult mortality (A), low child and low adult mortality (B), low child and high adult mortality (C), high child and high adult mortality (D), and high child and very high adult mortality (E). The division of regions into sub-regions resulted in 14 mortality sub-regions, a complete list of which (including the constituent countries) is provided in Appendix I [see Additional file 1].

The measure of population health that was used was the disability adjusted life year (DALY) [26]. This was used in preference to other measures of health, because it was conceptually clearer to think about redistributing DALY's between populations than, say, the mortality rate. The DALY is a measure of years of life lost attributable to morbidity and mortality within a population and has been used by the WHO [18] and some national governments [27] as a measure of the burden of disease. The measure of population wealth that was used was the gross national product (GNP) [28]. GNP is the broadest measure of national income and comprises gross domestic product plus net receipts of primary income from nonresident sources.

### Data

The data on the total number of DALYs and the size of the population of each mortality sub-region for 2000 was obtained from "GBD 2000 Version 1 Estimates" available from the WHO website. The DALY makes adjustments for age and sex at the time that death or disability occurred and a discount rate was also applied to the years of life lost.

The GNP for each of the mortality sub-regions was derived from the World Bank "World Development Indicators – 1999" CD-ROM, following adjustment for the purchasing power of an "international dollar" in each sub-region [28]. The 2000 GNP was estimated by multiplying each sub-region's population for 2000 by the relevant 1999 GNP per capita. The use of a purchasing power parity (PPP) measure overcame, to some degree, the undervaluing of consumption in economies with relatively low prices and the overvaluing of consumption in countries with high prices.

Where GNP figures were unavailable for a country within a mortality sub-region, the median GNP per capita for the remaining countries was used to estimate the missing country's GNP. A sensitivity analysis revealed that there was little difference in the overall results whether missing country data were imputed on the basis of a sub-region's median, mean, maximum or minimum GNP per capita.

### Analysis

The analysis is based on the decomposition of an index of inequality known as the Pietra Ratio [29], or Robin Hood Index [30]. The index was so named because in the context of wealth inequality it approximates the share of wealth that has to be transferred from those with greater than the mean-level resources and given to those with less than mean-level resources in order to achieve equality. In effect it is the share of the wealth that has to be robbed from the rich and given to the poor. In the context of this article, health inequality represents the share of the burden of disease as measured by the DALY, that needs to be transferred from the regions with the worst per capita health and transferred to the regions with the best per capita health in order to achieve health equality.

Although the Pietra Ratio is a single number reflecting the amount of wealth (or in this case, health) that needs to be transferred to achieve equality; rather than a single global index one can provide a decomposition of the ratio, indicating the extent to which each member of the population (in this case WHO sub-regions) needs to transfer wealth (or health) to ensure equality.

It is easiest to describe the approach to the analysis with reference to real data, and this is done as the article progresses.

## Results & discussion

Globally, in 2000 there were 0.247 DALYs per capita. That is, a quarter of a DALY had been lost for every living person. Regionally, the distribution was uneven with AFROE (0.635 DALYs per capita) and AFROD (0.502 DALYs per capita) showing the worst population health outcomes, and WPROA (0.107 DALYs per capita) and EUROA (0.129 DALYs per capita) showing the best population health outcomes.

**Figure 1:**
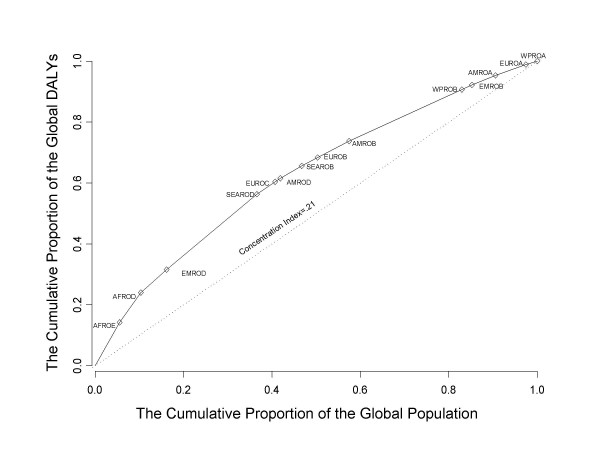
The Lorenz curve of the global distribution of health (DALYs) by WHO mortality sub-region.

The magnitude of the is demonstrable in a number of ways, with the Lorenz curve being one of the better known illustrations (Figure 1). The curve is fitted directly to the data points and hence the lack of deviation. Having ordered the WHO sub-regions according to their DALYs per capita burden, the Lorenz curve shows the proportion of global DALYs that are accounted for, by any given proportion of the world's population. Under conditions of equality (i.e., DALYs per capita are equal for each mortality sub-region), one would observe 10% of the DALYs accounted for by 10% of the world's population, 20% of the DALYs accounted for by 20% of the world's population, and so on. This line of equality appears as the straight (dotted) line. The degree to which the actual data (the solid line) deviate from the line of equality illustrates the degree to which poor heath is unequally distributed across the mortality sub-regions.

The inequality in the geographical distribution of the burden of disease is clear. The African region (the AFROE and AFDROD sub-regions combined), for example, accounts for about 10% of the world's population. It also accounts for about 24% of the global DALYs; i.e., 2.4 times the burden of disease that would be expected under conditions of equality. The inclusion of one of the Eastern Mediterranean mortality sub-regions (EMROD) shows that around 16% of the world's population accounts for around 32% of the global DALYs, still twice as large as would be expected under conditions of equality. Progressively, the inclusion of more regions sees the magnitude in the inequality decline. This pattern of inequality in distribution is entirely in keeping with expectation given that WPROA, the sub-region with the best health outcomes, includes countries like Australia, New Zealand, and Japan, while AFROE, the sub-region with the worst health outcomes, includes countries like South Africa, Burundi, Mozambique, and Rwanda.

If the magnitude of the inequality is assumed to correspond to the magnitude of the inequity, then one way to consider the problem of health equity is to ask the following. If one were to redistribute the DALYs in such a way that each sub-region bore the same per capita burden of disease (i.e., health were distributed equally), how would that redistribution manifest itself? This invites a form of counterfactual analysis similar to one used by Allotey and Reidpath to examine the global distribution of refugees [31].

Given that the global average DALYs per capita in 2000 were 0.247, by multiplying each sub-regions population by the global average the "expected" number of DALYs in each sub-region under conditions of equality can be calculated. The WHO sub-region AFROE, for example, had a population of approximately 330 million people in 2000. If health were distributed equally across sub-regions, one would expect AFROE to have experienced 81.5 million DALYs. The actual DALYs for the sub-region were approximately 209.6 million DALYs. In the monetary language of the Pietra Ratio, AFROE would have to reduc its burden to 40% of its actual level to achieve health equality. The ratio of the expected and the actual (or observed) DALYs per capita in each sub-region indicates the magnitude of the increase or decrease in the burden of disease that would be required in that sub-region for equality in the distribution of health to be achieved.

**Figure 2:**
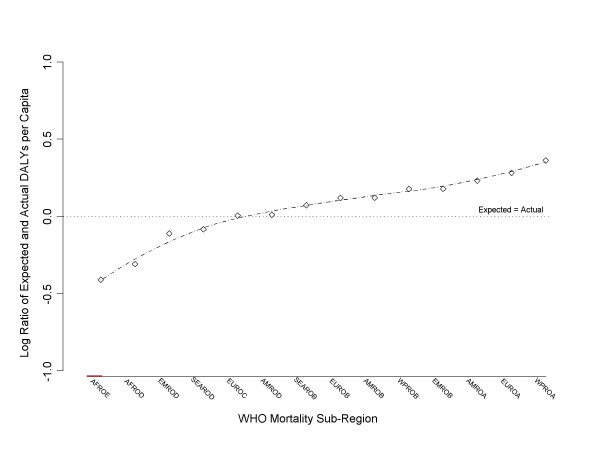
The log ratio of expected to actual per capita health (DALYs) illustrating the inequality of health distribution by WHO mortality sub-region.

Figure 2 shows the plot of the log ratio of expected and actual DALYs per capita for each of the 14 mortality sub-regions. A loess regression line is plotted against the points. A horizontal, reference line that assumes pre-existing equality is also plotted at 0 on the y-axis. Deviations are minimized because the data points (mortality sub-regions) are pre-ordered from low to high (left to right).

The percentage decrease in the burden of disease required by those regions lying below the line of equality – or the percentage increase in the burden of disease required by those regions lying above the line of equality – can be estimated from the graph by calculating the *10*^*x *^value of a region's log-ratio on the right-hand axis. For equality in the distribution of global health to be achieved the two African sub-regions would need to reduce their burden of disease to around 40% (AFROE) and 50% (AFROD) of their respective levels – that is, *10*^-*0.41 *^and *10*^-*0.31*^. EMROD would need to reduce its burden to around 80% of its level, as would SEAROD. EUROC and AMROD experienced very close to the burden of disease expected under conditions of global equality. All the other regions would be expected to increase their burden of disease. WPROB EMROB would need to increase the burden of disease by 50%. AMROA would need to increase the burden by 70%, EUROA by 90%, and WPROA by 230%!

Stark as the results are, however, they represent an analysis of health inequality, with a presumption that the magnitude of the inequality corresponds to the magnitude of inequity. As argued earlier, however, the unfairness (i.e., inequity) inherent in the distribution of health is not simply one of inequality. Even were health as measured by the DALY distributed equally across the sub-regions, the true burden would remain unequally distributed. It is also the wealth of sub-regions that allows them to mitigate the impact of morbidity and mortality on individuals, households, and society. *Ceteris paribus*, for any given per capita level of DALY, the negative impact of poor health will be less in a wealthier sub-region than in a poorer sub-region [22,23]. An analysis of health equity, rather than simply health inequality, would need to take this into account. In order to ascertain the magnitude of a health inequity, the question needs to be rephrased to realize the advantage that wealth offers a mortality sub-region in coping with the burden of disease. Specifically, given two regions with the same size population, one with a per capita GNP twice that of the other, one may "expect" under conditions of equity that the wealthier region would bear a greater burden of disease – and to simplify the illustration we will assume that it would bear a greater burden in direct proportion to its greater wealth – that is, twice the burden.

Thus, instead of assuming that it would be equitable for each sub-region to achieve identical DALYs per capita (i.e., .247 per capita), it is assumed that each region should achieve a level of DALYs per capita in proportion to its per capita wealth. The sub-region WPROA has a per capita wealth of $23,685 and the sub-region AFROE has a per capita wealth $1802. Under this form of equity, WPROA would be expected to experience a per capita DALYs level of 13.14 times that of AFROE. Table 1 shows the DALYs per capita and the GNP per capita for each of the mortality sub-regions.

**Table 1:** The distribution of health (DALYs), wealth (GNP) and population across the 14 WHO mortality sub-regions.

**Region**	**Sub-Region**	**Population (1,000,000)**	**GNP (USD$ PPP) ($1,000,000)**	**DALYs (1,000,000)**	**GNP per Capita**	**DALYs per Capita**
AFRO	E	330	594660	210	1802	0.635
AFRO	D	286	412309	144	1442	0.502
EMRO	D	348	672567	111	1933	0.319
SEARO	D	1219	1939586	365	1591	0.299
EURO	C	246	954966	60	3882	0.244
AMRO	D	70	262236	17	3746	0.240
SEARO	B	289	1156093	60	4000	0.209
EURO	B	215	1182823	40	5502	0.187
AMRO	B	425	3082915	80	7254	0.187
WPRO	B	1521	5158738	249	3392	0.164
EMRO	B	137	825521	22	6026	0.164
AMRO	A	318	9019217	46	28362	0.145
EURO	A	410	8095845	53	19746	0.129
WPRO	A	153	3623750	16	23685	0.107

**Global**		**5967**	**36981225**	**1472**	**6198**	**0.247**

**Figure 3:**
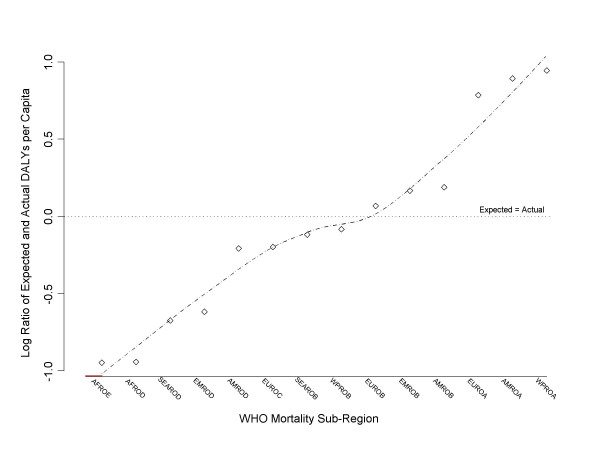
The log ratio of expected to actual per capita health (DALYs) weighted by per capita wealth (GNP) illustrating the inequity of health distribution by WHO mortality sub-region.

The counterfactual world of equitably distributed DALYs is created by redistributing all 1472 million DALYs in such a way that each sub-region's "expected" DALYs per capita stand in the same ratio to each other as their GNP per capita. Following the same general approach as before, Figure 3 shows the plot of the log ratio of expected and actual DALYs per capita for each of the 14 mortality sub-regions given a GNP adjusted distribution of the burden of disease. The scale on the y-axis is the same as in Figure 2, and again, a loess regression line is plotted, as is a reference line assuming pre-existing equality. There is slightly greater observable deviation than in Figure 3 – an artifact of the data.

The most striking feature of the figure is that the gradient of the loess line is generally much steeper than the equivalent line in Figure 2, indicating that the inequity embodied in the distribution of health is far greater than one would imagine if overcoming inequity was seen as the logical end-point of health policy. Under an equitable model of health redistribution the African region (both AFROE and AFROD) should carry about 10% of the burden of disease that it currently carries. EMROD and SEAROD should carry only 20% of the burden that they currently carry, and EUROC and AMROD would carry only 70% of the burden that they currently carry. On the other hand EUROA would have to support 600% of the burden that it currently supports, AMROA would have to support almost 800% and WPROA would have to support almost 900%!

The least healthy WHO mortality sub-region, AFROE, is around four times worse off under a health inequity analysis than would be revealed under a straight health inequality analysis. The healthiest sub-region, WPROA, is about four times better off. This suggests that an analysis based on the inequality of health substantially undercounts the magnitude of the health inequity.

One might construct an argument on the basis of these results indicating that the way to achieve an equitable distribution of health would be to increase the health burden in those healthier populations. This is a completely logical consequence of any measure (such as this one) which is based around an underlying measure of inequality; because inequality can always be reduced by decreasing the advantage of those who are most advantaged. At the time of the analysis there were 1472 million DALYs to be redistributed. Increasing the health burden of the healthiest populations would by its very nature improve the health equity of this measure, but it would be completely undesirable to increase the global burden of disease. More cancer for the USA, Canada, and Australia is not the strategy of choice.

Measures of an inequity do not necessarily embody the appropriate policy intervention, and it would be a mistake to assume that they do or that they should. An alternative and more palatable approach to reducing the magnitude of the inequity would be to improve the health and wealth of the poorest and most burdened populations of the world.

Before concluding, there are some possible limitations associated with the analysis. The two most significant limitations relate to the regional nature of the analysis, and the approach used to weight the data by regional wealth. Regions, or more particularly WHO mortality sub-regions, are not homogenous. Countries within regions can differ markedly from one to another. For instance, Cuba and the United States, two radically different national entities, are included in the same sub-region in virtue of their mortality profile, but obviously without regard to their wealth. Sub-region aggregation, however, is a feature of current DALYs data, which are not available at the country level. Although country level data would be preferable, the association between per capita wealth and per capita health at the sub-region level is broadly similar to what is known about the association at the national and sub-national level. The results of the analysis, therefore, are not an artifact of the regional aggregation of data.

There is also a question about the appropriateness of the wealth weighting function as a compensatory device for translating a health inequality measure into a health inequity measure. The equity analysis assumes that every additional dollar per capita has a constant effect on the encumbrance of poor health. The extent to which this assumption is reasonable requires further investigation and alternative weighting functions could be applied. It is unlikely, however, that more dollars per capita would ever be a bad thing in the context of the equity weighting. Under these circumstances the thrust of the present findings would remain, although the ultimate shape of the wealth weighting function becomes a question for further investigation.

## Conclusion

An analysis of health equity requires more than the identification of socially determined health inequality, or the measurement of the magnitude of the inequality. The encumbrances of poor health, that is, the direct burden of the disease and the broader social and economic costs are affected by factors such as the quality and availability of supportive physical infrastructure, healthcare services, and social services. Wealthy regions enjoy better physical infrastructure, healthcare services, and social services, all of which mitigate the impact of poor health [22,23]. Because these mitigators are functions of societies' wealth, in an analysis of health equity, wealth needs to be factored in to the measurement process.

The inequity of poor health experienced by poorer regions around the world is significantly worse than a simple analysis of health inequality reveals. By measuring the inequity and not simply the inequality, the magnitude of the disparity can be factored into future economic and health policy decision making.

## Supplementary Material

Additional file 1 WHO subregions. A list of the 14 WHO mortality regions, sub-regions, mortality profiles, and member states. Click here for file
