# Supplementary material for: Measuring global health inequity
Source: Int J Equity Health. 2007 Oct 30;6:16. doi: 10.1186/1475-9276-6-16 (PMC2147004; doi:10.1186/1475-9276-6-16)
Supplement: Additional file 1 — WHO subregions. A list of the 14 WHO mortality regions, sub-regions, mortality profiles, and member states. [file 1475-9276-6-16-S1.pdf]

WHO Regions and mortality subregions used in epidemiological analyses and reporting for the World Health Report 2000[1].

Region	Sub-Region	Mortality Type	WHO Member State
AFRO	D	High Child<br>High Adult	Algeria, Angola, Benin, Burkina Faso, Cameroon, Cape Verde, Chad, Comoros, Equatorial Guinea, Gabon, Gambia, Ghana, Guinea, Guinea-Bissau, Liberia, Madagascar, Mali, Mauritania, Mauritius, Niger, Nigeria, Sao Tome and Principe, Senegal, Seychelles, Sierra Leone, Togo
AFRO	E	High Child<br>Very High Adult	Botswana, Burundi, Central African Republic, Congo, Côte d'Ivoire, Democratic Republic of Congo, Eritrea, Ethiopia, Kenya, Lesotho, Malawi, Mozambique, Namibia, Rwanda, South Africa, Swaziland, Uganda, United Republic of Tanzania, Zambia, Zimbabwe
AMRO	A	Very Low Child<br>Low Adult	Canada, Cuba, United States of America
AMRO	B	Low Child<br>Low Adult	Antigua and Barbuda, Argentina, Bahamas, Barbados, Belize, Brazil, Chile, Colombia, Costa Rica, Dominica, Dominican Republic, El Salvador, Grenada, Guyana, Honduras, Jamaica, Mexico, Panama, Paraguay, Saint Kitts and Nevis, Saint Lucia, Saint Vincent and the Grenadines, Suriname, Trinidad and Tobago, Uruguay, Venezuela
AMRO	D	High Child<br>High Adult	Bolivia, Ecuador, Guatemala, Haiti, Nicaragua, Peru
EMRO	B	Low Child<br>Low Adult	Bahrain, Cyprus, Iran (Islamic Republic of), Jordan, Kuwait, Lebanon, Libyan Arab Jamahiriya, Oman, Qatar, Saudi Arabia, Syrian Arab Republic, Tunisia, United Arab Emirates
EMRO	D	High Child<br>High Adult	Afghanistan, Azerbaijan, Djibouti, Egypt, Iraq, Morocco, Pakistan, Somalia, Sudan, Yemen
EURO	A	Very Low Child<br>Low Adult	Andorra, Austria, Belgium, Croatia, Czech Republic, Denmark, Finland, France, Germany, Greece, Iceland, Ireland, Israel, Italy, Luxembourg, Malta, Monaco, Netherlands, Norway, Portugal, San Marino, Slovenia, Spain, Sweden, Switzerland, United Kingdom
EURO	B	Low Child<br>Low Adult	Albania, Armenia, Bosnia and Herzegovina, Bulgaria, Georgia, Kyrgyzstan, Poland, Romania, Slovakia, Tajikistan, The Former Yugoslav Republic of Macedonia, Turkey, Turkmenistan, Uzbekistan, Yugoslavia
EURO	C	Low child<br>High adult	Belarus, Estonia, Hungary, Kazakhstan, Latvia, Lithuania, Republic of Moldova, Russian Federation, Ukraine
SEARO	B	Low child<br>Low adult	Indonesia, Sri Lanka, Thailand
SEARO	D	High child<br>High adult	Bangladesh, Bhutan, Democratic People's Republic of Korea, India, Maldives, Myanmar, Nepal
WPRO	A	Very low child<br>Low adult	Australia, Brunei Darussalam, Japan, New Zealand, Singapore
WPRO	B	Low Child<br>Low Adult	Cambodia, China, Cook Islands, Fiji, Kiribati, Lao People's Democratic Republic, Malaysia, Marshall Islands, Micronesia (Federated States of), Mongolia, Nauru, Niue, Palau, Papua New Guinea, Philippines, Republic of Korea, Samoa, Solomon Islands, Tonga, Tuvalu, Vanuatu, Viet Nam

1 Mathers CD, Sadana R, Salomon JA, Murray CJL, Lopez AD (2000). Estimates of DALE for 191 countries: methods and results. *Global Programme on Evidence for Health Policy Working Paper No. 16*. Geneva: World Health Organization
